# Comparing Mental Health of School-Age Children with and without Epilepsy

**Published:** 2016

**Authors:** Farshid SHAMSAEI, Fatemeh CHERAGHI, Gholamreza ZAMANI

**Affiliations:** 1 Mother and Child Care Health Center, Hamadan University of Medical sciences, Hamadan, Iran; 2 Chronic Diseases (home care) Research Center, Hamadan University of Medical sciences, Hamadan, Iran; 3 Children’s Medical Center, Tehran University of Medical Sciences, Iranian epilepsy association board, Tehran, Iran

**Keywords:** Mental Health, Child, Epilepsy

## Abstract

**Objective:**

Mental health problems frequently occur in children with epilepsy but the diagnosis is frequently missed and therapeutic opportunities are often lost. The aim of this study was to compare mental health statues between school-aged children with epilepsy and the healthy group.

**Materials & Methods:**

In this case, control study, 120 children aged 6 to 12 years with idiopathic epilepsy and 240 healthy control groups were followed up. Children with epilepsy were enrolled from Iranian Epilepsy Association in 2014. The parent version of Child Symptom Inventory-4 questionnaire was used. Mean comparisons were performed using Student’s t test while effect sizes were estimated by Cohen’s d coefficient. The Chi-Square test was used to assess the difference between frequency distribution of demographic variables in both groups. The significance level was considered less than 0.05.

**Results:**

There were statistically significant differences between children with epilepsy and control group as for attention deficit hyperactivity disorder, generalized anxiety disorder, major depression, separation anxiety, social phobia, motor and vocal tics and oppositional defiant disorder.

**Conclusion:**

The carefully evaluating and prospectively following the psychopathology symptom of children with epilepsy are critical for early identification, prevention and treatment.

## Introduction

Epilepsy is a one of the most common neurological disorders of childhood leading to excessive and abnormal neuronal activity in the brain (1). Epilepsy disorders affect the brain and cause recurring seizures. In the United States, more than 450,000 children suffer from a type of epilepsy (2). The incidence of childhood epilepsy was 82.2/100000 children, markedly higher than that of the overall population (3). In Iran, the prevalence of epilepsy had increased from 2 to 34/100000 persons in 1970-2000 (4). Between children and adolescents with chronic diseases, the rates of mental disorders in those with epilepsy are higher than the other ones or even found in normal children. Psychological complications are generally associated withpediatric epilepsy (5). Besides, the prevalence of behavioral disorders in children with epilepsy is a two- or three times higher than in healthy children (6). The rates of psychiatric disorder were 37% in children and adolescents with epilepsy compared with 11% in diabetic children of 5-15 yr-old (7). Mood changes, anxiety and psychosis, attention deficit hyperactivity disorder (ADHD) and autism are relatively frequent comorbidities of children and adolescents with epilepsy. In general, these psychiatric disorders were known as the complications of the seizure disorders. However now, a bidirectional relationship has been confirmed (8, 9). Depressive, anxiety disorders and attention-deficit-hyperactivity disorder were elevated in children with epilepsy (10). Even though the prevalence of psychiatric disorders is high, these results were based on research tools that can be influenced by seizure phenomenology, clinical samples and the efforts for causal analysis (8). As a result of the impact of psychiatric disorders on the routine of daily life of children and adolescents with epilepsy, further studies is necessary for better understanding of their mental health and appropriated treatment of these comorbidities. Besides, despite the high levels of psychiatric comorbidity with epilepsy, many children and adolescents with epileptic disorders that suffered from psychiatric illness do not receive early enough attention and adequate treatment (6). The aim of the current study was to compare mental health disorders of school-age children with epilepsy and in a healthy group.

## Materials & Methods

**Study design and setting**

This case–control study was conducted from August to December 2014, in Iranian Epilepsy Association. The sample size included 120 children with epilepsy. Inclusion criteria were diagnosis of epilepsy and idiopathic epileptic syndrome established according to the clinician neurologist diagnostic examination, age 6-12 yr, with regular attendance at school, no mental retardation and chronic physical diseases of child, negative family history of any diagnostic mental disorders in parents and sibling, and living in Hamadan City, western Iran. The subjects were selectedby convenience sampling and then every child with epilepsy was matched for age, gender, education and birth order with two healthy controls.

**Instruments**

Two questionnaires included demographic and Child Symptom Inventory-4 (CSI-4) was used to collect data. Demographic questionnaire included children’s personal variables such as age, gender, birth order, education and parents personal variables such as age, gender, educational degree, marital status, occupation, history of mental illness, history of psychiatric medication use or any other diseases. The case and control groups were matched in terms of demographic variables. To assess children’s mental health disorders, CSI-4 was used. This inventory is a behavior rating scale designed by Sprafkin and Gadow to screen behavioral and affecting disorders in children aged 5 to 12 yr (11). CSI-4 has two forms for parents and for teachers; we used the former in the present study. CSI-4 is a DSM-IV-referenced rating scale that screens for affecting and behavioral symptoms of childhood psychiatric disorders. There are both parent (97 items) and teacher versions (77 items). The CSI-4: Parent Checklist contains screens for 15 affecting and behavioral disorders, and the CSI-4: Teacher Checklist contains screens for 13 affecting and behavioral disorders (12). The CSI-4 can be scored to obtain symptom count scores or symptom severity scores. Validity and reliability of the parent form of the questionnaire were assessed in Iranian children and results showed appropriate validity and reliability (13). Questionnaires were completed as self-report by one of the parents in the case and control groups after they met the inclusion criteria and were matched for demographic variables.

**Ethics**

The Hamadan University of Medical sciences Ethics Committee approved thet study. Written informed consent was obtained from the subjects. It is also noteworthy that the results of the study were anonymously reported to comply with the ethical criteria.

**Statistical analysis**

Data were analyzed using descriptive statistics of frequency, percentage, mean, standard deviation, and inferential statistics in SPSS software version 18 (Chicago, IL, USA). Kolmogorov-Smirnov test was used to evaluate the normal distribution of the quantitative data. Assuming the normality of data collected, independent t-test was used to compare the mean scores of the two groups, otherwise U MannWhitney was used. The Chi-Square test was used to assess the significant difference between frequency distribution of demographic variables in both groups. The significance level was less than 0.05.

## Results

In terms of demographic characteristics, the mean age of children was 11.8 yr, 55.8% were boys, and 42.5% were the first child and were in the four grades (Table 1). Independent t-test results showed that there was a significant difference between the mean score of GSI- 4 among children with epilepsy and children in the control group in terms of attention deficit hyperactivity disorder, generalized anxiety disorder, major depression, separation anxiety (P<0.001), social phobia, motor and vocal tics and oppositional defiant disorder (P<0.05). In other words, the mean scores of these disorders were more in the case group. Using the Mann-Whitney test, there was also a significant difference between the case and control groups in terms of obsessive thoughts, compulsive activity, dysthymic disorder, conduct disorder. Therefore, the mean of these disorders was higher in the case group (Table 2).

## Discussion

Attention to mental health of children is of special importance. As a high-risk group, children with epilepsy should be noted to detect and screen their early and prodromal symptoms of any the mental disorders. It helps to carry out and prompt nessesery interventions. Our findings showed that the mean scores on the subscales of attention deficit hyperactivity disorder, generalized anxiety disorder, major depression, separation anxiety and social phobia in children with epilepsy were higher than in controls group.Our findings are consistent with other similar studies where attention-deficit/hyperactivity disorder was the most common psychiatric comorbidity associated with pediatric epilepsy and the prevalence ranged from 20% to 38% depending upon assessment methods and samples (14). In the present study, despite the previous ones, prevalence of depression ranged from 26% to 33% and mean score of depression and anxiety in children with epilepsy group was high. In another study that evaluated psychiatric comorbidity in children with epilepsy, the psychiatric disorders such as anxiety, depression, behavioral disorders and social phobias in these patients were significantly higher than healthy group (15). Williams et al. found similar disorders in subscales of anxiety, depression and aggressive behaviors (16). Similar to present study, the results of their studies indicated higher mean score of depression and anxiety in children with epilepsy. An increased risk of depression and anxiety among patients with epilepsy suffering from more than one type of the psychiatric comorbidity disorders is reported (17). Children with epilepsy less than twelve aged old tend to have higher scores from the CBCL questionnaire, in total, internalizing and externalizing problems (18). Similar the finding of present study, McDermott et al. reported that rates of mental disorders in children and adolescents with epilepsy were higher than in normal children and also in those with other chronic diseases (19). Furthermore, the prevalence of behavioral disorders in children with epilepsy was 4.7 times higher than healthy children (20). The results of the present study also indicate higher mean score of attention deficit hyperactivity disorder (ADHD) children with epilepsy. Similar to present study, parisi et al. reported that attention deficit hyperactivity disorder was more frequent in children with epilepsy than in general pediatric population (21). Several factors may contribute to it such as the underlying brain pathology, the types of epilepsies, the chronic consequences of seizure and the antiepileptic drugs effects. However, in epileptic children with ADHD, treatment might become a challenge for child neurologists, treatment with psychotropic drugs can be safely in most of them (21). Living with epilepsy is one of the importance causesof mental health problems such as lack of access to adequate confidence, conduct disorder, anxiety, hyperactivity and depression among children and a resaon for attempted suicide in adolescences and youths. Behavioral disorders of a person with epilepsy, the underlying tensions and conflicts unattainable ideals imposed on the one hand and the inability to realize the ideals becomes.

**Table 1:** Characteristics of Children with Epilepsy and Healthy Group

**Variables**	**Cases group**	**Control group**	*P*.value
**Sex**	N (%)	N (%)	
Female Male	53 (44.2) 67 (55.8)	105 (43.75) 135 (56.25)	X^2 ^=0.056 P=0.65
Birth Order			
1 2 3 4≥	51 (42.5) 30 (25) 23 (19.2) 16 (13.3)	101 (42.1) 60 (25) 46 (19.2) 33 (13.75)	Z=1.44 P= 0.3
Education Level			
1 2 3 4 5 6	13 (10.8) 15 (12.5) 22 (18.3) 31 (25.8) 18 (13.3) 21 (17.5)	26 (10.8) 31 (12.9) 44 (18.3) 63 (26.3) 34 (28.3) 42 (17.5)	X^2 ^=3.42 P= 0.8
**Age (yr)**	**Mean ±SD**	**Mean (SD)**	T= 0.41
	11.8 ±1.8	12.2 (2.1)	P=0.83

**Table 2:** Compression of Child Symptom Inventory-between Children with Epilepsy and Healthy Group

**Disorders**	**Cases**	**Controls**	**Test**	***P*** **. value**
	**Mean ± SD**	**Mean ± SD**		
Attention Deficit/ hyper activity disorder	43.7 ± 11.6	19.7 (5.1)	T=8.76	<.001
Oppositional defiant disorder	19.4 ± 4.9	17.3 (3.8)	T=7.8	<0.05
Conduct disorder	18.2 (4.2)	15.6 (1.1)	Z=-5.33	<0.05
Generalized anxiety disorder	18.7 (5.4)	11.5 (3.6)	T=6.74	<.001
Social phobia	9.7 (2.2)	7.6 (1.6)	T=3.22	<0.05
Separation anxiety disorder	19.6 (6.4)	14.9 (4.7)	T=4.97	<.001
Obsessive compulsive disorder	1.71 (0.8)	1.29 (0.4)	Z=-2.32	<0.05
Special phobia	2.5 (1.6)	1.1 (0.7)	T=3.23	<0.05
Post-traumatic stress disorder	1.5 (0.7)	1.2 (0.4)	Z=-4.5	<0.06
Major depression	10.5 (3.8)	7.7 (1.1)	T= 5.5	<0.001
Dysthymic Disorder	8.7 (1.43)	9.7 (0.53)	Z= -5.2	<0.05
Schizophrenia	4.8 (2.3)	4.5 (1.9)	T= 2.4	<0.07
Pervasive Developmental Disorder	18.7 (5.7)	14.8 (3.1)	T= 5.3	<0.001
Motor Tics	1.9 (1.49)	0.7 (0.59)	T=3.22	<0.05
Vocal Tics	1.8 (1.61)	0.5 (0.48)	T=2.92	<0.05

**Clinical Implications**

Findings indicate that emotional and behavioral problems of school-aged children with seizures should be assessed regularly during treatment and followup of child behaviors in the clinical settings. When children have many behavior problems, they should be referred to mental health professionals for counseling. All efforts should be made to prevent these children’s problems from escalating, because if they do, other family members’ behaviors could be affected. Support groups with other children who had seizures and their parents will provide opportunities to talk about their fears and concerns. The analysis of psychiatric impacts of children with epilepsy provide basic data required for making decisions, future research and generation of interventional strategies, all geared to promote comprehensive caring. Based on the child assessment, family interventional programs should be planned for promotion coping strategies and interpersonal skills in children with epilepsy and their relatives. An important component of comprehensive care is the availability of mental health professionals to assess and treat behavior problems found in children with seizures. The Epilepsy Associations are needed to develop and conduct supportive programs designed to help these children and their parents meet the challenges of raising a child with seizures.

**Limitations**

One important limitation was that seizure variables related to behavioral and emotional problems were not included in the analysis. Among the other limitations of this study is the reliance on parents as the only source of information about a child’s mental health status. Another limitation of this study is the cross-sectionalnature and the sample size was modest, meaning that a larger number of cases would be necessary in order to facilitate a more thorough statistical analysis of the psychopathological profiles. By applying CSI-4 questionnaire, it is possible to screen and categorize behavioral and emotional disorders used to guide prevention, diagnosis and maybe early treatment; however, it does not define the specific type of psychopathology. Other studies using the CSI-4 prospectively are suggested to find that children with epilepsy present high rates of psychiatric disorders and behavior problems, and that those rates remain high over time. In conclusion, the results reinforce the importance of studying mental health of children and adolescents with epilepsy. Epilepsy as a complex medical condition has a high prevalence in pediatrics population. In these children and adolescents psychiatric comorbidity is very high and in some ones may be need more attention and treatment than the seizure disorder itself. Children with epilepsy are at higher risk for having emotional, behavioral or mental health problems than other children especially those ones with chronic diseases. The probability rate of the mental health problems in these children and adolescents is higher than the general childhood population; therefore the need for more comprehensive management, beyond seizure control, with the aim to improve the quality of life and to reduce the limiting effects that the epilepsy can cause is highlighted. It is important that the mental health or social service professionals working with mentally ill adults find out about the children and adolescents’ mental health and emotional development too. Qualified mental health professionals can help and evaluate the mental status of these children using routine screening, counseling, and psychosocial support for cognitive and emotional conditions. Counseling therapy and social support groups for children and adolescents of living with epilepsy and also their families should be recommended. Health education, psychosocial development and better access to mental health services may also be helpful.
